# Role of N6-Methyladenosine Methylation Regulators in the Drug Therapy of Digestive System Tumours

**DOI:** 10.3389/fphar.2022.908079

**Published:** 2022-06-09

**Authors:** Zhelin Xia, Fanhua Kong, Kunpeng Wang, Xin Zhang

**Affiliations:** ^1^ Department of Pharmacy, Taizhou Central Hospital (Taizhou University Hospital), Taizhou, China; ^2^ Zhongnan Hospital of Wuhan University, Institute of Hepatobiliary Diseases of Wuhan University, Transplant Center of Wuhan University, National Quality Control Center for Donated Organ Procurement, Hubei Key Laboratory of Medical Technology on Transplantation, Hubei Clinical Research Center for Natural Polymer Biological Liver, Hubei Engineering Center of Natural Polymer-based Medical Materials, Wuhan, China; ^3^ Department of General Surgery Taizhou Central Hospital (Taizhou University, Hospital), Taizhou, China

**Keywords:** digestive system tumors, N6-methyladenosine, drug resistance, chemotherapy, immunotherapy

## Abstract

Digestive system tumours, including stomach, colon, esophagus, liver and pancreatic tumours, are serious diseases affecting human health. Although surgical treatment and postoperative chemoradiotherapy effectively improve patient survival, current diagnostic and therapeutic strategies for digestive system tumours lack sensitivity and specificity. Moreover, the tumour’s tolerance to drug therapy is enhanced owing to tumour cell heterogeneity. Thus, primary or acquired treatment resistance is currently the main hindrance to chemotherapy efficiency. N6-methyladenosine (m6A) has various biological functions in RNA modification. m6A modification, a key regulator of transcription expression, regulates RNA metabolism and biological processes through the interaction of m6A methyltransferase (“writers”) and demethylase (“erasers”) with the binding protein decoding m6A methylation (“readers”). Additionally, m6A modification regulates the occurrence and development of tumours and is a potential driving factor of tumour drug resistance. This review systematically summarises the regulatory mechanisms of m6A modification in the drug therapy of digestive system malignancies. Furthermore, it clarifies the related mechanisms and therapeutic prospects of m6A modification in the resistence of digestive system malignancies to drug therapy.

## Introduction

Digestive system tumours predominantly include stomach, colon, esophagus, liver and pancreatic tumours. Currently, these tumours have high morbidity and mortality rates. Among patients with digestive system tumours, the elderly account for approximately 68.5% (Sung et al., 2021). Surgery, including open, laparoscopic and endoscopic surgeries, is currently the standard treatment for digestive system tumours (Chesney et al., 2021). To date, several treatment strategies, including chemotherapy and radiation, have enhanced the disease-free and overall survival rates of patients with cancer (Zhang et al., 2020a). However, owing to the heterogeneity of cancer cells, primary or acquired treatment resistance is often observed, leading to treatment failure (Dagogo-Jack and Shaw, 2018). Additionally, the occurrence and development of digestive system tumours are reported to be related to the activation of oncogenes, inactivation of tumour suppressor genes and activation of abnormal cell signalling pathways. Furthermore, epigenetic processes regulate gene expression *via* DNA methylation, histone modification and RNA modification, thereby affecting the occurrence and development of tumours in the digestive system (Kong et al., 2020a).

Liver cancer is the fourth most common cause of cancer-related death worldwide, with hepatocellular carcinoma (HCC) as the most prevalent form, and the incidence of liver cancer is reported to be increasing globally (Kong et al., 2019; Chen et al., 2021a; Kong et al., 2021). HCC is the second most common cause of cancer-related death, but its current treatment strategies and outcomes are poor (Chen et al., 2021a; Llovet et al., 2021). Several drugs, such as Sorafenib (SOR) and Lenvatinib, have been approved for the first-line systemic therapy of advanced or unresectable patients with HCC (Al-Salama et al., 2019; Kant et al., 2021). Thus, the identification of new drug targets for the treatment of HCC is important, especially in tumour immuno-targeted therapy. Currently, systematic therapies, including immune checkpoint inhibitors (ICIs), tyrosine kinase inhibitors and monoclonal antibodies, have been reporting better outcomes than traditional HCC therapies (Llovet et al., 2021). Over the past five years, significant advances have been made in overall survival and the quality of life (Llovet et al., 2018). However, various challenges continue to exist in the treatment of HCC, such as drug resistance.

Pancreatic cancer, a highly lethal malignancy with a 5-year survival rate of approximately 10% in the United States, is becoming an increasingly common cause of cancer-associated death (Mizrahi et al., 2020). Currently, surgical resection remains the only option to cure pancreatic cancer. However, adjuvant chemotherapy has made significant progress in improving the prognosis of patients with pancreatic cancer (Wang et al., 2021a). Adjuvant chemotherapy regimens include FOLFIRINOX [5-fluorouracil (FU), folate, irinotecan and oxaliplatin (OX)] and gemcitabine combined with sodium protein, sodium and paclitaxel and have been shown to prolong overall survival (Mizrahi et al., 2020). Currently, many clinical trials are evaluating the effectiveness of immunotherapy strategies in pancreatic cancer, including ICIs; cancer vaccines; adoptive cell metastasis; and combinations with other immunotherapy agents, chemoradiotherapy, or other molecule-targeted agents. However, the therapeutic outcomes of these strategies remain poor (Schizas et al., 2020). Therefore, the molecular mechanism of pancreatic cancer requires further exploration.

Colorectal cancer (CRC) is the third most common cancer type and has the third-highest rate of cancer-related deaths in the United States (Kong et al., 2020b). CRC ranks 2nd to 4th in the global incidence of cancer depending on region, cancer type or gender (Sawicki et al., 2021). It remains one of the deadliest diseases worldwide due to the lack of early detection methods and appropriate drug treatment strategies (Pavitra et al., 2021). Surgery remains the preferred treatment method for CRC (Chen et al., 2018). Additionally, chemotherapy has become an effective treatment to prolong the survival of patients with CRC. Currently, the commonly used treatment regimens are FOLFOX (OX + calcium folate and FU), CAPEOX (OX + capecitabine) and FOLFIRI (irinotecan + calcium folate and FU) (Dekker et al., 2019). However, the treatment and prognosis of CRC have been unsatisfactory, especially for patients with metastasis. Therefore, the identification of novel drug targets and the improvement of drug resistance could effectively improve the prognosis and survival rate of patients with CRC.

Gastric cancer is the fifth most common cancer worldwide and the third most common cause of cancer-associated deaths (Smyth et al., 2020). Gastric cancer is a multifactorial disease wherein environmental and genetic factors influence its occurrence and development. It is also a highly invasive and heterogeneous malignant tumour (Machlowska et al., 2020). Presently, surgery is the first-line of treatment for gastric cancer (Johnston and Beckman, 2019). Postoperative adjuvant radiotherapy/chemotherapy and targeted therapy have become a routine course of treatment for gastric cancer. Furthermore, active early screening could effectively aid in the early diagnosis of gastric cancer. However, the early diagnosis of gastric cancer remains a challenge due to the poor specificity of diagnostic markers and the cost of screening (Johnston and Beckman, 2019). Therefore, the development of new diagnostic and therapeutic targets is vital to the treatment of gastric cancer.

N6-methyladenosine (m6A) is considered to be the most common, abundant and conserved internal transcriptional modification, especially in eukaryotic messenger RNA (mRNA) (Huo et al., 2020). m6A modification exists in mRNA and various non-coding RNAs (Ma et al., 2019; Huang et al., 2020). m6A is modified by m6A methyltransferase (writer), removed by m6A methylase (eraser) and recognized by reading proteins (reader). It regulates RNA metabolism, including translation, splicing, export, degradation and microRNA (miRNA) processing. Recently, m6A RNA modification has been proved to play a key role in tumour development (Yan et al., 2021). The alteration of m6A levels regulates the expression of tumour-related genes, such as BRD4, MYC, SOCS2 and epidermal growth factor receptor (EGFR), thereby promoting the pathogenesis and development of tumours (He et al., 2019). Many studies report that the dysregulation of m6A is associated with the progression and drug resistance of various cancers, suggesting that m6A regulatory factors can be used as therapeutic targets in cancer treatment and biomarkers in overcoming drug resistance (Xu et al., 2020a).

## Molecular Mechanisms of N6-Methyladenosine Modification

### N6-Methyladenosine Writers

**FIGURE 1:**
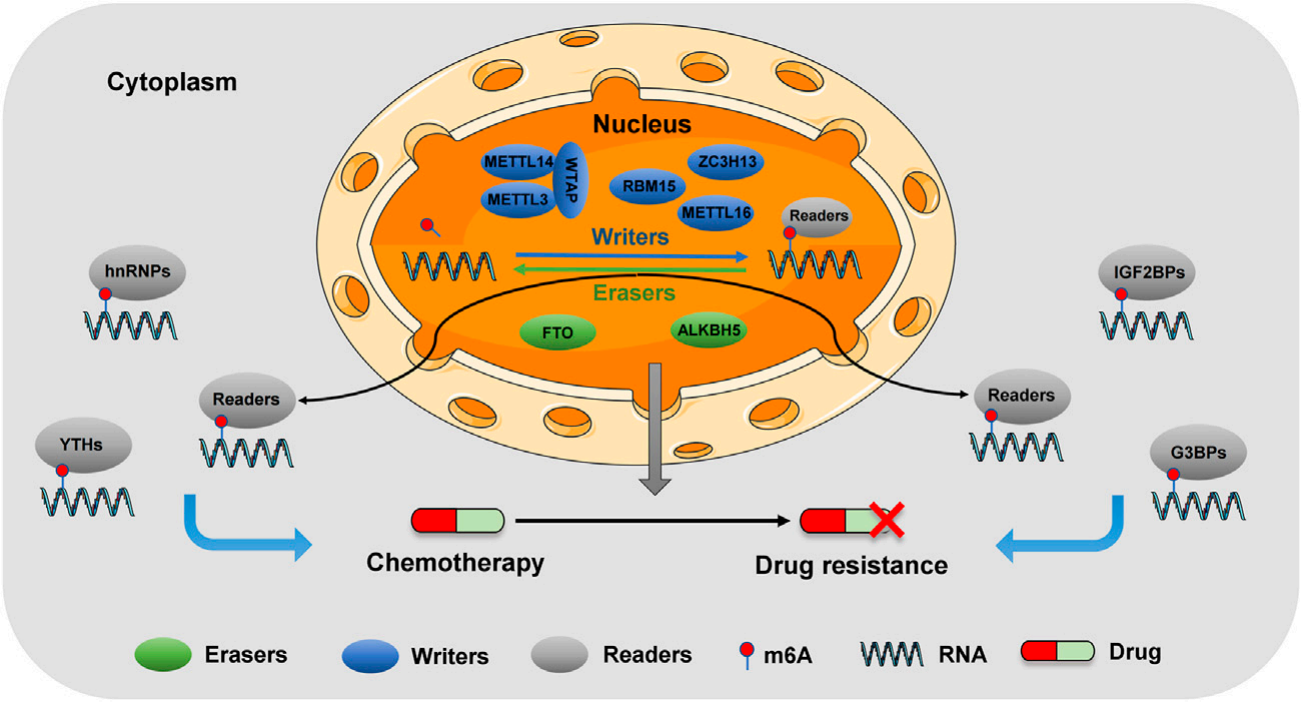
Molecular mechanisms of m6A modification in digestive tumors. m6A modification is a dynamic and reversible process. Methyltransferase complexes (writers) catalyze m6A methylation, demethylase (erasers) reverse m6A methylation, and m6A binding protein (readers) promote its function. m6A methylation is involved in carcinogenesis and chemotherapy resistance of digestive tumors.

m6A writers are composed of KIAA1429 (VIRMA), METTL3, RBM15, WTAP, ZC3H13, METTL16, METTL14, and CBLL1 (Shen et al., 2020). As a heterodimer, METTL3/METTL14 can be catalysed and bound by WTAP, which interacts with METTL3/METTL14 and regulates the translation stability of mRNA (Schöller et al., 2018). KIAA1429 plays a key role in guiding the deposition of regionally selective m6A (Hu et al., 2020) and regulating the expression of sex-lethal genes by the selective splicing of pre-mRNA using WTAP (Bansal et al., 2014). METTL16, a newly discovered RNA m6A methyltransferase, acts as an RNA-binding protein (RBP) and plays a key role in SAM homeostasis by regulating SAM synthase MAT2A mRNA (Doxtader et al., 2018). RBM15, an RBP, can regulate Notch, Wnt and other signalling pathways and affect the development of various tumour cells (Wang et al., 2021b). ZC3H13 plays the role of a tumour suppressor, mainly inhibiting tumour occurrence by regulating the Ras–ERK signalling pathway (Zhu et al., 2019a). The mechanism of action of m6A writers in tumours is shown in Figure 1.

### N6-Methyladenosine Erasers

m6A erasers are predominantly composed of ALKBH5 and FTO (Huo et al., 2020). ALKBH5 regulates RNA metabolism through m6A demethylation, such as pre-mRNA processing and mRNA decay and translation (Qu et al., 2022). It participates in the modification of oncogene or tumour suppressor gene mRNA in an m6A-dependent manner. Moreover, ALKBH5 regulates the transcriptome of tumours, causing changes in cell proliferation, survival, invasion and metastasis; drug sensitivity; tumour stem cell status; and tumour immunity (Qu et al., 2022). Given that ALKBH5 has high substrate specificity in tumours, targeting ALKBH5 has promising potential in cancer treatment (Wang et al., 2020a). On understanding ALKBH5 structure, mediated carcinogenesis and drug reaction mechanism, ALKBH5-targeted therapy could be applied in clinical practice. FTO, a demethylase, was originally identified to be involved in the development of obesity and type 2 diabetes. This gene encodes the FTO protein, which belongs to the ALKB dioxygenase family that is dependent on Fe^2+^ and 2-oxoglutarate (Gerken et al., 2007). The dysregulation of FTO demethylation has been identified as a driver of various diseases, including cancer, metabolic diseases and neuropsychiatric disorders (Annapoorna et al., 2019). Studies have found that the abnormal expression of FTO is increasingly associated with various diseases, especially cancer. Thus, the development of FTO modulators has potential therapeutic applications. Recent studies report that inhibitors that interfere with FTO activity show significant therapeutic effects in different cancers, thus providing a new strategy for identifying drugs that target external transcriptomic RNA methylation in drug discovery (Zhou et al., 2021). The mechanism of action of m6A erasers in tumours is illustrated in Figure 1.

### N6-Methyladenosine Readers

m6A readers can be divided into three types based on binding m6A-containing transcription: YTH domain (YTH family protein), HNRNP family (hnRNPC, hnRNPG and hnRNPA2B1) and common RNA-binding domain and its flanking regions (IGF2BPs and hnRNPA2B1) (Shi et al., 2021; Huang et al., 2022). Additionally, FXR family, IGF2BP family, eIF family and G3BPs family (44). Heterogeneous nuclear ribonucleoproteins (hnRNPs) are a large family of RBPs that are involved in the many aspects of nucleic acid metabolism, including alternative splicing, mRNA stabilization and transcription and translation regulation (Geuens et al., 2016). hnRNP family proteins are abnormally expressed in most tumours and play a role in promoting tumour occurrence and development. The YTH domain protein family is the main “reader” of m6A modification while the YTH domain can recognize and bind m6A-containing RNA. YTH family proteins have different functions to determine the metabolic fate of m6A-modified RNAs (Shi et al., 2021). YTHDF1 selectively recognises m6A-modified mRNA through the YTH domain, promotes its loading into ribosomes and interacts with initiation factors to promote its translation through the N-terminal domain (Wang et al., 2015). Conversely, YTHDF2 selectively binds m6A-modified RNA and regulates its degradation by recruiting CCR4–NOT complexes to accelerate RNA de-enylation (Zhao et al., 2017). YTHDF3 acts as an assigner, following which YTHDF1 and YTHDF2 competitively interact with YTHDF3, thus determining the fate of the mRNA transcript (Jin et al., 2020). YTHDC1, a widely expressed nuclear protein, is located in YT bodies near nuclear spots and phosphorylated by members of the SRC and TEC tyrosine kinase families in the cytoplasm, leading to its conversion function in RNA splicing (Rafalska et al., 2004). Like other proteins in the YTH family, YTHDC2 can recognize and bind to the m6A fragment of mRNA to play a regulatory role (Wojtas et al., 2017). YTHDC2 can improve the translation efficiency of its target, thus affecting the occurrence of tumours (Hsu et al., 2017). The mechanism of m6A readers in tumours is shown in Figure 1.

## Implications of N6-Methyladenosine in Cancer Chemotherapy

The prognosis of digestive tract tumours has been significantly improved in recent years. However, the early diagnosis of digestive tract tumours remains elusive, and the phenomenon of drug resistance persists. Moreover, the underlying aetiology of these malignancies remains unclear, therefore, the epigenetic factors that promote the occurrence and development of digestive malignancies should be elucidated and novel biomarkers or effective therapeutic targets should be simultaneously identified (Seebacher et al., 2019). Numerous studies report that m6A modification promotes the occurrence and development of tumours by regulating oncogene expression and inhibiting genes (Huo et al., 2020). Through epigenetic modification, m6A can promote and inhibit tumorigenesis, playing a “double-edged sword” role (He et al., 2019). Furthermore, the m6A regulatory protein is a therapeutic target for cancer and plays an important biological role in the resistance of malignant tumours to chemotherapy (Yu et al., 2019). Additionally, studies show that the mechanisms of drug resistance in malignant tumours are complex and diverse. m6A-dependent RNA modification has also received extensive attention as a potential determinant of tumour heterogeneity and chemotherapy response.

Presently, the identification of efficient and safe chemical drugs for m6A modification is under study. In particular, few drugs based on natural products are characterised by novel structures, various biological activities and reliable safety (Lu and Wang, 2020). Therefore, an m6A regulator based on natural product discovery is considered the future research direction. Furthermore, modern drug discovery platforms, which are characterized by a combination of web-based pharmacology, chemical databases derived from natural resources, computer-aided design and chemical modifications, have been recognized to aid in the development of new drugs that target m6A regulators (Zhang et al., 2021a). Recently, natural products have been used as the chemical libraries of m6A-targeted anticancer drugs, subsequently becoming potential anti-tumour drugs. For example, curcumin is a natural phenolic compound that down-regulates the expression of ALKBH5 and enhances the expression of m6A modified TRAF4-mRNA (Chen et al., 2021b). Resveratrol is a natural polyphenol with antioxidant, anti-inflammatory, heart-protective and anticancer properties that can be used in combination with curcumin to reduce m6A modifications, thereby effectively improving normal growth performance and intestinal mucosal integrity (Gan et al., 2019). Quercetin, another flavonoid, has various biological functions, including anti-cancer activity. It can inhibit the proliferation, migration and invasion of HeLa and SiHa cells synergistically with cisplatin by inhibiting METTL3 expression (Xu et al., 2021). Recent studies suggest that betaine plays an important role in the methylation of m6A. Zhang et al. found that betaine inhibited the expression of m6A methylases, METTL3 and METTL14, in HepG2 cells, but promoted the expression of demethylases, FTO and ALKBH5 Zhang et al. (2019). In addition to these natural products, other active natural products have also been shown to have anti-M6A bioactivity and anti-cancer activity. Fusarium acid decreases p53 expression in HCC HepG2 cells by down-regulating the m6A methylation of p53-mRNA (Ghazi et al., 2021). Although many studies have shown promising prospects for the development of targeted m6A modification drugs, only a few have potential drug-capabilities and can be used as therapeutic targets for cancer treatment. The currently developed m6A modification inhibitors and activators still have disadvantages, such as poor target specificity, efficacy, safety and pharmacokinetics (Huff et al., 2021). Hence, developing novel drugs is vital to cancer treatment.

Thanks to the rapid development of science and technology, especially artificial intelligence (AI) technology and computer technology, the newly discovered drugs have the advantages of fast speed, easy use and cost-saving. Presently, AI-assisted technology has been widely used in drug candidate discovery and development (Paul et al., 2021). Chen et al. (2012) developed a series of FTO inhibitors using AI techniques. The natural product rhein was identified as the first cell-based FTO inhibitor, which also inhibited ALKBH2 activity. Additionally, they designed and synthesised eight luciferin molecules whose structures were similar to two luciferin molecules. The structure-activity relationship of these fluorescent FTO inhibitors was elucidated by the X-ray crystal structure of FTO/luciferin complexes. These studies demonstrate advancement in identifying novel chemical CLASS FTO inhibitors with strictly defined physicochemical properties by combining structure-based drug design with high-throughput *in vitro* inhibition test systems. Additionally, Huang et al. identified Entacapone as an FTO inhibitor by combining several methods, including structure-based hierarchical virtual screening strategies, biochemical experiments, *in vivo* experiments and transcriptome sequencing analysis (Peng et al., 2019). Chen et al. also found two effective FTO inhibitors CS1 and CS2 using structure-based virtual screening (Su et al., 2020). Using molecular docking, Lan et al. identified a cage-like molecular activator of METTL3/14, the photocured substituent-linked MPCH. The drug activates METTL3/14 and results in m6A hypermethylation under short periods of ultraviolet light exposure (Lan et al., 2021a). Currently, AI has aided in the development of revolutionary approaches to drug discovery, design and development, therefore, targeting m6A modification regulators could be a possibility, thereby proving its therapeutic potential in cancer treatment.

## Effects of N6-Methyladenosine on Drugs in Tumour Therapy

### Hepatocellular Carcinoma

**FIGURE 2:**
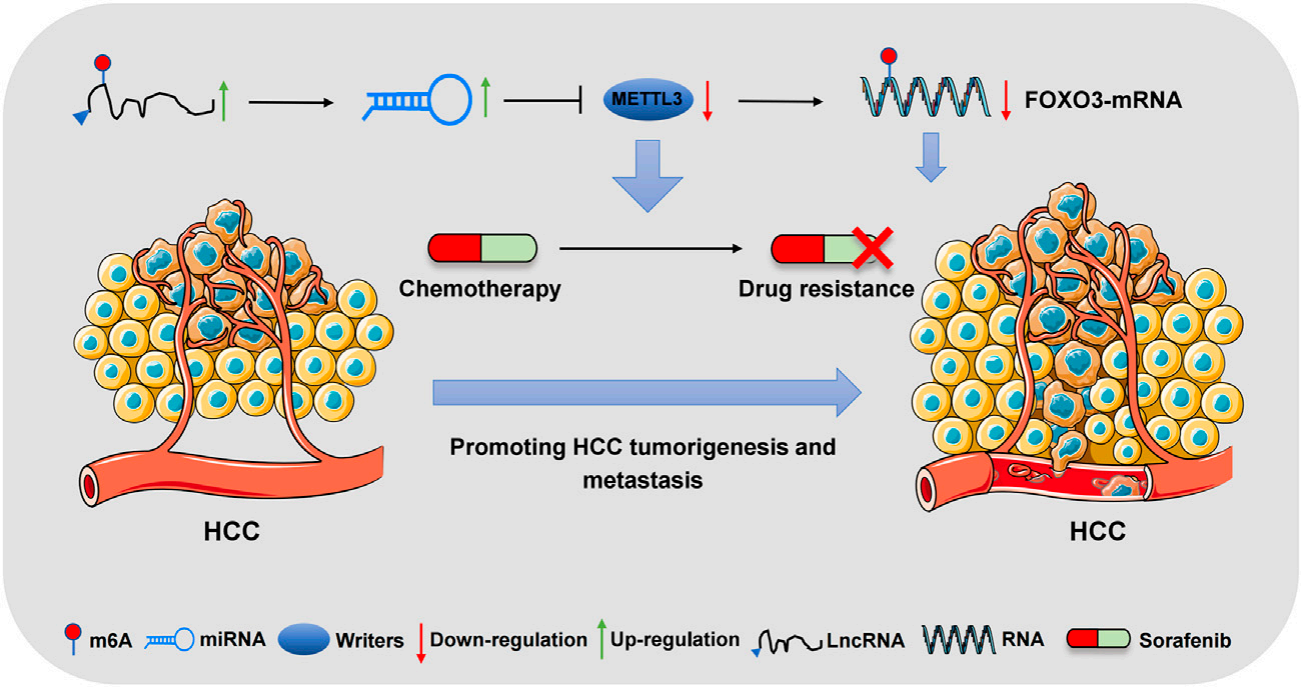
The regulatory mechanism of m6A modification on chemotherapy resistance in HCC. METTL3, as an m6A writer, is down-regulated in HCC and regulated by LncRNA and miRNA. Meanwhile, METTL3 also regulates the stability of FOXO-mRNA and promotes the resistance of HCC to sorafenib.

Liver cancer is the sixth most common cancer worldwide, including HCC and cholangiocarcinoma (CCA) subtypes. Recent studies have shown that m6A regulatory factor is closely related to the development of HCC and is expected to be a potential therapeutic target for HCC (Xu et al., 2019). Moreover, HCC is often too advanced for surgical treatment by the time it is diagnosed (Kong et al., 2022). SOR, a bisaryl urea multikinase inhibitor, is the first molecularly targeted drug approved by the Food and Drug Administration (FDA) for the clinical treatment of HCC (Kong et al., 2021). SOR has strong antitumour and antiangiogenic effects, effectively improving the survival rate of patients with advanced HCC (Palazzo et al., 2010; Iacovelli et al., 2015). However, during treatment, changes in epigenetic modifications occur due to the heterogeneity of HCC. This phenomenon suggests that acquired or primary SOR resistance is a major obstacle to the survival of patients with HCC (Zhu et al., 2017a). As an important member of RNA modification, m6A modification plays an important role in regulating drug resistance during HCC treatment. As the most famous m6A methyltransferase, METTL3 has been identified as a key regulator in many biological processes, including cell cycle, apoptosis, migration, invasion, differentiation and inflammatory response (Maldonado López and Capell, 2021). Kong et al. (2022) showed that the lncRNA LINC01273 can promote the resistance of HCC to SOR. LINC01273 increases the stability of miR-600 by acting as a “reservoir” and enhances the inhibition of miR-600 on METTL3 mRNA, resulting in the downregulation of METTL3 and drug resistance of HCC cells to SOR. Additionally, METTL3 increases the m6A level of LINC01273 and decreases the stability of LINC01273 in recognizing YTHDF2. Therefore, the dysregulation of the LINC01273/miR-600/METTL3 axis could be a potential cause of SOR resistance in HCC cells. Notably, METTL3 is often reported as an oncogene and is upregulated in most tumours (Zeng et al., 2020). This could be associated with the dual regulatory role of the m6A regulatory factor. Lin et al. (2020) also found that METTL3 expression is significantly downregulated in SOR-resistant HCC cells. The deletion or depletion of METTL3 promotes the expression of SOR drug resistance and angiogenesis genes and activates autophagy-related pathways. The downregulation of METTL3 expression leads to the decreased stability of FOXO3-mRNA, which promotes the resistance of HCC to SOR. This indicates that METTL3 is a negative regulator of SOR resistance and could be related to the bidirectional action of METTL3, and its internal mechanism is worth further study. Additionally, Xu et al. (2020b) showed that m6A modification promotes the drug resistance of HCC to SOR by regulating the expression level of circRNA-SORE, especially *via* an m6A modification site in circRNA-SORE. Furthermore, the m6A level of circRNA-SORE was increased in SOR-resistant HepG2 cells and the level of circRNA-SORE was significantly decreased on METTL3/14 knockout. These results indicated that m6A modification promotes HCC resistance to SOR through the circRNA expression level. Currently, SOR has become the first-line drug for patients with advanced liver cancer. Despite the wide use of SOR, it has certain disadvantages in its clinical application (Llovet et al., 2008; Cervello et al., 2012). Some patients acquire resistance to SOR in the course of treatment, which affects the overall survival time of patients with HCC (Zhu et al., 2017b). Therefore, further studies on the mechanism of drug resistance could improve and overcome this obstacle. The mechanism of m6A regulatory factors in HCC is shown in Figure 2 and Table 1.

**TABLE 1:** The roles of different m6A regulators in hepatocellular carcinoma.

m6A regulators	Genes/RNAs	Drugs	Mechanism	Function	References
METTL3	LncRNA LINC01273, miR-600	Sorafenib	1. Enhance the inhibitory effect of miR-600 on METTL3	Increased resistance to Sorafenib	Kong et al. (2022)
			2. Down-regulation of METTL3		
YTHDF2	LncRNA LINC01273	Sorafenib	1. METTL3 increases the m6A level of LINC01273	Increased resistance to Sorafenib	Kong et al. (2022)
			2. Decreased the stability of LINC01273 in recognizing YTHDF2		
METTL3	FOXO3-mRNA	Sorafenib	1. Down-regulation of METTL3	Increased resistance to Sorafenib	Lin et al. (2020)
			2. Decreased the stability of FOXO3-mRNA		
METTL3/14	CircRNA-SORE	Sorafenib	m6A modification of circRNA-SORE was increased	Increased resistance to Sorafenib	Xu et al. (2020b)

### Pancreatic Cancer

Pancreatic cancer is one of the leading causes of cancer-related deaths in the Western world, owing to its advanced nature, early metastasis and limited response to chemotherapy or radiation. Adjuvant chemotherapy after surgical resection is the preferred treatment for early pancreatic cancer (Zeng et al., 2019). Although gemcitabine remains a cornerstone in the treatment of early-stage advanced pancreatic cancer, its clinical efficacy is poor due to molecular mechanisms, epigenetic modifications, limitations in cell uptake and activation and chemotherapeutic resistance development within weeks of treatment initiation (Amrutkar and Gladhaug, 2017). Current studies on the mechanism of drug resistance in pancreatic cancer report that m6A modification plays an important role in the drug resistance of pancreatic cancer to chemotherapy drugs. The deletion of METTL3, an m6A writer, enhances the sensitivity of pancreatic cancer cells to gemcitabine, 5-FU, cisplatin and radiotherapy. Furthermore, METTL3 could promote the resistance of pancreatic cancer to gemcitabine, 5-FU and cisplatin via several key pathways, including the MAPK cascade, ubiquitin-dependent processes, RNA splicing and cellular process regulation (Taketo et al., 2018). METTL14, another regulator of m6A writer, forms a functional heterodimer with METTL3, which is further catalysed and stabilised by WTAP, promoting the effect of m6A modification (Schöller et al., 2018). Furthermore, METTL14, one of the key methyltransferases, is an RNA-binding scaffold that recognizes the substrate of the m6A methyltransferase complex and has 20% sequence homology with METTL3. Among m6A methyltransferases, METTL14 is speculated to have a methyltransferase function that aids in RNA binding and METTL3 stabilisation (Wang et al., 2017). Kong et al. (2020a) showed that METTL14 is upregulated in pancreatic cancer tissues, with METTL14 knockdown in pancreatic cancer cells enhancing its sensitivity to cisplatin therapy. METTL14 regulates the sensitivity of pancreatic cancer cells to cisplatin treatment via the AMPKα, ERK1/2, and mTOR signalling pathways and improves autophagy via the mTOR signalling pathway. Additionally, METTL14 expression is closely associated with gemcitabine-resistant treatment and upregulated in gemcitabine-resistant human pancreatic cancer cells. METTL14 increases the expression of cytidine deaminase, an enzyme that inhibits gemcitabine. Therefore, METTL14 knockdown significantly increases the sensitivity of gemcitabine in drug-resistant cells (Zhang et al., 2021b). Although the expression of METTL14 is increased in drug-resistant pancreatic cancer cells, its expression regulation mechanism remains unclear. Therefore, further studies on the mechanism of METTL14 resistance are required.

**FIGURE 3:**
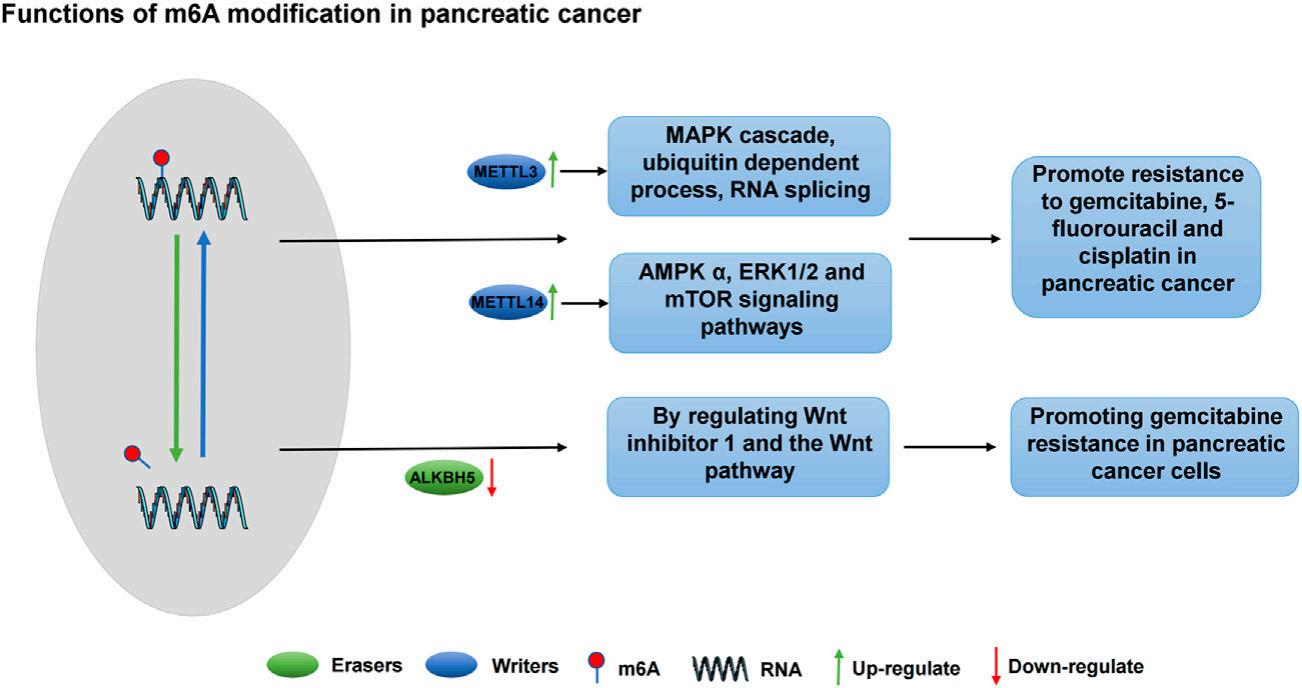
The regulatory mechanism of m6A modification on chemotherapy resistance in pancreatic cancer. METTL3 and METTL14 are up-regulated in pancreatic cancer and promote drug resistance to gemcitabine, 5-fluorouracil and cisplatin through MAPK cascade, ubiquitin-dependent process, RNA splicing, AMPK α, ERK1/2, and mTOR signaling pathways. The down-regulated expression of ALKBH5 in pancreatic cancer affects the sensitivity of pancreatic cancer cells to gemcitabine by regulating Wnt inhibitor 1 and the Wnt pathway.

ALKBH5, an m6A eraser, is downregulated in pancreatic cancer. The overexpression of ALKBH5 can inhibit the proliferation, migration and invasive activities of pancreatic cancer (Guo et al., 2020). Compared with existing tumour markers, ALKBH5 shows good prognostic ability, and its expression level is positively correlated with the prognosis of The Cancer Genome Atlas cohort patients (Cho et al., 2018). In patients with pancreatic cancer treated with gemcitabine, ALKBH5 expression is down regulated, whereas its overexpression induces the pancreatic cancer cells’ sensitivity to chemotherapy. The sensitivity of pancreatic cancer cells to gemcitabine is affected by the regulation of Wnt inhibitor 1 and the Wnt pathway (Tang et al., 2020). Unlike readers and writers, only two m6A demethylases, FTO and ALKBH5, are known that rely on Fe (II) and α-ketoglutaric acid (Dai et al., 2018). The downregulation of m6A promotes the resistance of FTO and ALKBH5 to PARPi. Moreover, m6A was confirmed to play an important regulatory role in treatments related to DNA damage response, including radiotherapy, chemotherapy and therapy targeting mutations related to DNA damage repair. Considering that the crystal structures of FTO and ALKBH5 have been determined, the development of drugs targeting FTO and ALKBH5 is a potential research direction (Han et al., 2010; Aik et al., 2014). The mechanism of the m6A regulatory factor in pancreatic cancer is shown in Figure 3 and Table 2.

**TABLE 2:** The roles of different m6A regulators in pancreatic cancer.

m6A regulators	Genes/RNAs	Drugs	Mechanism	Function	References
METTL3	MAPK cascades	Gemcitabine	Up-regulation of METTL3	Increased resistance to gemcitabine, 5-fluorouracil and cis-platinum	Taketo et al. (2018)
		5-fluorouracil			
		Cis-platinum			
METTL14	--	Cis-platinum	1.Up-regulation of METTL14	Increased resistance to cis-platinum	Kong et al. (2020a)
			2. Through AMPK α, ERK1/2 and mTOR signaling pathways		
METTL14	Cytidine deaminase (CDA)	Gemcitabine	1. Up-regulation of METTL14	Increased resistance to gemcitabine	Zhang et al. (2021b)
			2. Increased cytidine deaminase (CDA) expression		
ALKBH5	Wnt inhibitory factor 1 (WIF1)	Gemcitabine	1. Down-regulation of ALKBH5	Increased resistance to gemcitabine	Tang et al. (2020)
			2. Regulation of Wnt inhibitory factor 1 and Wnt pathway		

### Colorectal Cancer

Radical surgical resection is the preferred treatment regimen for CRC, and radiotherapy and chemotherapy, as a routine treatment strategy after surgery, can effectively improve the survival of patients (Liu et al., 2019; Kong et al., 2020b). OX, a third-generation platinum drug, is widely used as a first-line chemotherapy agent for CRC. However, repeated long-term dosing induces chemotherapeutic resistance by increasing the expression of multidrug-resistant proteins, glutathione and excision repair cross-complement and promoting cell export and excision repair nucleotides (Allen and Johnston, 2005). Lan et al. (2021b) found that total m6A RNA content and critical methyltransferase METTL3 expression were increased in the CRC tissues of patients with OX resistance. The overexpression of METTL3 enhances the resistance of CRC cells to OX *via* TRAF5-mediated necrosis. Patients with CRC often develop resistance to 5-FU. Studies showed that miRNAs in exosomes secreted by cancer-associated fibroblasts (CAFs) are associated with sensitivity to 5-FU (Hu et al., 2019). METTL3 promotes miR-181d-5p secretion through the DiGeorge Syndrome Critical Region 8 (DGCR8) in CAFs. CAF-derived exosomes enhance 5-FU resistance in CRC cells *via* the METTL3/miR-181d-5p axis. Thus, a novel role of exosome miR-181d-5p secreted by CAFs was revealed. METTL3-dependent m6A methylation is upregulated in CRC, thereby promoting miR-181d-5p processing by DGCR8, resulting in increased miR-181d-5plevels, and inhibiting 5-FU sensitivity by targeting NCALD (Pan et al., 2022). METTL3 also promotes CRC resistance to 5-FU through circ-0000677 (Liu et al., 2022a). Additionally, METTL3 catalyses transition adenosine methylation and promotes pre-mRNA preferential splicing and p53 protein’s R273H mutation, leading to acquired drug resistance in colon cancer cells (Uddin et al., 2019).

Cisplatin is a platinum-based chemotherapy drug that has been clinically proven to treat various malignant tumours (Dasari and Tchounwou, 2014). Although cisplatin therapy has achieved good prognosis and survival rates in patients with cancer, problems of drug resistance and considerable side effects remain (Galluzzi et al., 2012). YTHDF1, an m6A “reader”, is an important regulator of tumour progression (Chen et al., 2021c). The expression of YTHDF1 is significantly upregulated in CRC, and its overexpression can reduce the sensitivity of colon cancer cells to cisplatin. YTHDF1 promotes the synthesis of GLS1 protein by binding to GLS1 3′UTR, which causes cisplatin resistance in colon cancer cells (Chen et al., 2021d). Yang et al. (2021) analysed CRC cells using proteomic and transcriptomic analyses to identify proteins involved in multidrug resistance in CRC. Results showed that IGF2BP3 expression was upregulated in CRC, whereas IGF2BP3 knockout significantly improved the sensitivity of CRC to adriamycin. Therefore, IGF2BP3 could be a potential biomarker to predict the occurrence of CRC multidrug resistance. Targeting IGF2BP3 could also be a potential chemotherapy strategy to prevent the development of multidrug resistance in CRC. FTO, an m6A eraser, blocks the ability of cancer stem cells through its N-6,2′-O-dimethyladenosine demethylase activity. The downregulation of FTO expression in CRC enhances the m6A modification level of mRNA, leading to increased tumorigenicity and chemotherapy resistance *in vivo* (Relier et al., 2021).

**FIGURE 4:**
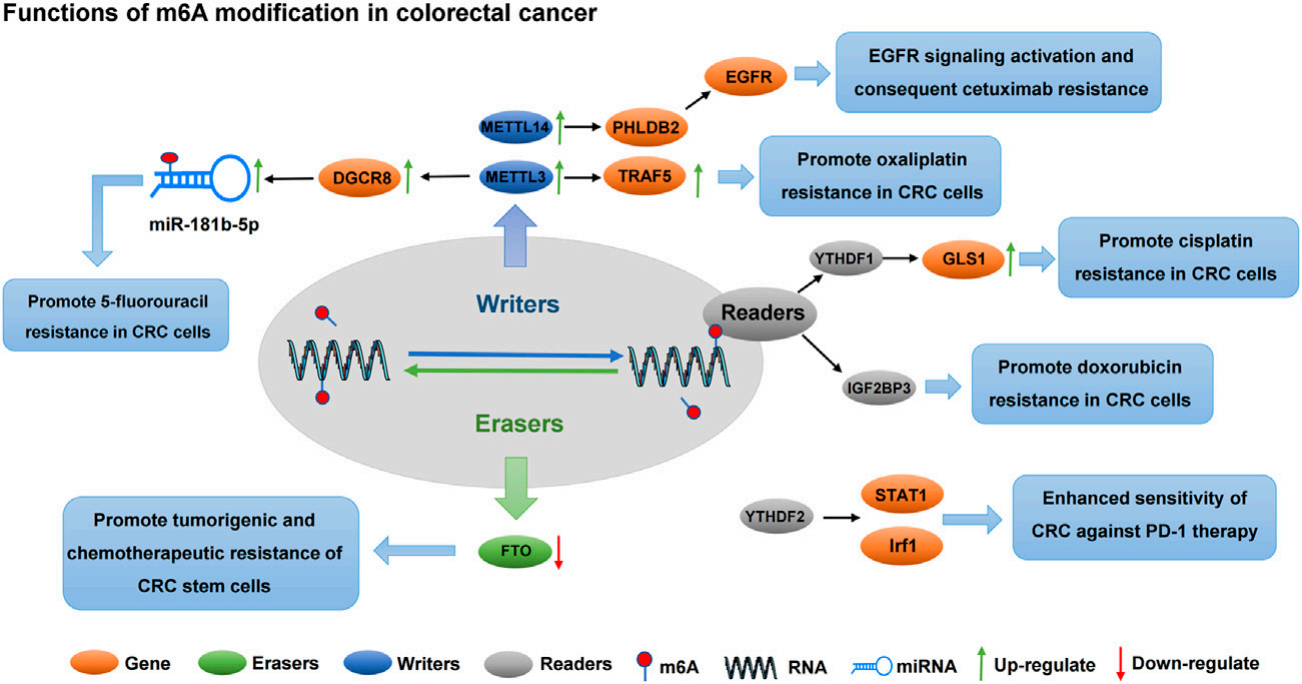
The regulatory mechanism of m6A modification on chemotherapy resistance in CRC. In CRC, m6A Writers, Erasers, and Readers are dysregulated and promote CRC resistance to cisplatin, 5-fluorouracil, oxaliplatin, doxorubicin, and targeted agents by regulating the stability of downstream gene expression.

Immunotherapy and targeted therapy are current strategies for the treatment of CRC. Programmed cell death-1 (PD-1) checkpoint blocking immunotherapy has achieved impressive clinical success in treating various cancers (Lipson et al., 2015). However, limited or nonresponsive to PD-1 antibody therapy remains a challenge (Ganesh et al., 2019). In immunotherapy-resistant CRC, the deletion of METTL3 and METTL14 enhances the response of CRC and melanoma to PD-1 therapy. This could be attributed to the downregulated expression of METTL3 or METTL14, YTHDF2-stabilized STAT1 and Irf1 mRNA, activated IFN-γ–STAT1–IRF1 signalling pathway and enhanced sensitivity of CRC against PD-1 treatment (Wang et al., 2020b). Cetuximab is an FDA-approved monoclonal antibody against EGFR that is recommended for patients with metastatic CRC and wild-type KRAS/NRAS/BRAF tumours; however, its efficacy remains unsatisfactory, especially in patients with potentially metastatic CRC and adjuvant therapy progression (Benson et al., 2021). Additionally, PHLDB2 is upregulated in CRC and promotes the migration and invasion of cancer cells. METTL14 regulates PHLDB2 and promotes its expression, whereas PHLDB2 upregulation stabilises EGFR and promotes its nuclear translocation, leading to EGFR signal transduction activation and cetuximab resistance (Luo et al., 2022). Therefore, PHLDB2 is a potential therapeutic target for CRC. Hao et al. report that MIR100HG expression is closely related to the markers of epithelial–mesenchymal transformation in CRC and can serve as a positive regulator of epithelial–mesenchymal transformation. MIR100HG maintains cetuximab resistance *in vitro* and *in vivo* and promotes the invasion and metastasis of CRC cells. Furthermore, hnRNPA2B1 binds with MIR100HG and maintains the mRNA stability of TCF7L2. hnRNPA2B1 identifies the TCF7L2 mRNA m6A site *via* the MIR100HG hnRNPA2B1/TCF7L2 axis and enhances CRC resistance to cetuximab (Liu et al., 2022b). Therefore, targeted therapy combined with MIR100HG and immune checkpoint blockade could be a potential therapeutic strategy to improve the immunotherapy response of patients with CRC. The mechanism of m6A regulation in CRC is shown in Figure 4 and Table 3.

**TABLE 3:** The roles of different m6A regulators in colorectal cancer.

m6A regulators	Genes/RNAs	Drugs	Mechanism	Function	References
METTL3	TRAF5-mRNA	Oxaliplatin	1. Up-regulation of METTL3	Increased resistance to oxaliplatin	Lan et al. (2021b)
			2. Regulation of TRAF5 expression		
METTL3	DGCR8-mRNA miR-181b-5p	5-fluorouracil	METTL3 promotes the secretion of miR-181b-5p by DGCR8	Increased resistance to 5-fluorouracil	Pan et al. (2022)
METTL3	circ_0000677 ABCC1-Mrna	5-fluorouracil	1. METTL3 enhances the m6A level of CIRC_0000677	Increased resistance to 5-fluorouracil	Liu et al. (2022a)
			2. Circ_0000677 regulates ABCC1 and promotes CRC resistance		
YTHDF1	GLS1-mRNA	Cis-platinum	1. Up-regulation of YTHDF1	Increased resistance to cis-platinum	Chen et al. (2021d)
			2. Promote the synthesis of GLS1 protein		
IGF2BP3	ABCB1-mRNA	Doxorubicin	1. Up-regulation of IGF2BP3	Increased resistance to doxorubicin	Yang et al. (2021)
			2. IGF2BP3 promotes ABCB1 expression		
FTO	PCIF1/CAPAM	5-fluorouracil	Down-regulation of FTO	Increased resistance to 5-fluorouracil	Relier et al. (2021)
METTL3/14	STAT1-mRNA	Anti-PD-1 antibody	1.Up-regulation of METTL3/14	Increased resistance to Anti-PD-1 antibody	Wang et al. (2020b)
	Irf1-mRNA		2. Activation of IFN-γ -STAT1-IRF1 signaling pathway		
METTL14	PHLDB2-mRNA	Cetuximab	1. METTL14 promotes PHLDB2 expression	Increased resistance to Cetuximab	Luo et al. (2022)
			PHLDB		
			2. Activates EGFR signal transduction		
hnRNPA2B1	MIR100HG-mRNA	Cetuximab	Activate MIR100HG/hnRNPA2B1/TCF7L2 axis	Increased resistance to Cetuximab	Liu et al. (2022b)
	TCF7L2-mRNA				

### Gastric Cancer

Endoscopic resection is the standard treatment regimen for early gastric cancer. Non-inchoate operable gastric cancer is treated by surgery while sequential chemotherapy is used for advanced gastric cancer. The first-line treatment also includes platinum drugs and FU double chain (Smyth et al., 2020). OX, a first-line treatment for advanced gastric cancer, has been widely used in clinical settings; however, drug resistance mainly causes treatment failure (Boku et al., 2019; Harada et al., 2021). Li et al. (2022a) showed that CD133+ stem cell-like cells are the main subgroup of OX resistance whereas PARP1 is the central gene mediating OX resistance in gastric cancer. PARP1 can effectively repair the DNA damage caused by OX, leading to the occurrence of drug resistance. Moreover, METTL3 expression is upregulated in CD133+ stem cells. METTL3 recruits YTHDF1 to enhance the stability of PARP1 mRNA, thus playing a role in the repair of PARP1-mediated DNA damage and the development of OX resistance in gastric cancer cells. In addition to OX, cisplatin, either alone or in combination with other chemotherapeutic agents, is a first-line chemotherapy drug for patients with advanced gastric cancer (Kang et al., 2020). However, cisplatin resistance remains a major challenge in the treatment of advanced gastric cancer (Wang et al., 2016). Zhu et al. (2022) found that lncRNA LINC00942 is upregulated in gastric cancer and associated with poor prognosis. LINC00942 upregulates MSI2 expression by blocking the interaction between MSI2 and SCF^β−TrCP^ E3 ubiquitin ligase, ultimately inhibiting its ubiquitination. Subsequently, LINC00942 enhances C-Myc mRNA stability in an m6A-dependent manner and enhances cisplatin resistance in gastric cancer. Therefore, blocking the LINC00942–MSI2–C-Myc axis could be a novel therapeutic strategy for patients with chemotherapy-resistant gastric cancer. Another lncRNA, ARHGAP5-AS1, is upregulated in gastric cancer and associated with poor prognosis. ARHGAP5-AS1 enhances the stability of ARHGAP5 mRNA by recruiting METTL3 and modifying ARHGAP5 mRNA. Therefore, the upregulation of ARHGAP5 could promote cisplatin chemotherapy resistance in gastric cancer (Zhu et al., 2019b). Hence, targeting the ARHGAP5-AS1/ARHGAP5 axis is a promising strategy for overcoming chemotherapy resistance in gastric cancer. Recently, a special relationship between the tumour microenvironment infiltration of immune cells and m6A modification has been revealed, which cannot be explained by the mechanism of RNA degradation. Therefore, a comprehensive understanding of the characteristics of cell infiltration in the tumour microenvironment mediated by multiple m6A regulatory factors could aid in our understanding of the tumour microenvironment immune regulation. Zhang et al. (2020b) comprehensively analysed the m6A landscape associated with immunophenotype in 1,938 gastric cancer samples and constructed an m6A scoring system called the m6Ascore to quantify the m6A characteristics associated with immune cell infiltration in individual patients with GC. When the m6A score was low, the neoantigen load was increased and immune infiltration was high, indicating that the immune checkpoint blockade (PD-1 and PD-L1) has good clinical efficacy. Therefore, m6A modification plays an important role in the diversity and complexity of the tumour microenvironment. Additionally, Feng et al. (2021) speculate that proton pump inhibitors could be a promising therapeutic strategy to further improve the sensitivity of gastric cancer cells to antitumour drugs. For example, omeprazole pre-treatment can enhance the inhibitory effect of 5-FU, DDP and TAX on gastric cancer cells, increase the total m6A level of gastric cancer cells and inhibit autophagy, thereby improving the anti-tumour efficiency of chemotherapy drugs. The mechanism of m6A regulation in gastric cancer is shown in Table 4.

**TABLE 4:** The roles of different m6A regulators in gastric cancer.

m6A regulators	Genes/RNAs	Drugs	Mechanism	Function	References
METTL3 YTHDF1	PARP1- mRNA	Oxaliplatin	1. PARP1 repairs DNA damage caused by oxaliplatin	Increased resistance to oxaliplatin	Li et al. (2022a)
			2. METTL3 recruited YTHDF1 to enhance the stability of PARP1 mRNA		
METTL3-METTL14-WTAP complex	LncRNA LINC00942 c-Myc-mRNA	Cis-platinum	LINC00942 enhances the stability of c-Myc-mRNA in an m6A dependent manner	Increased resistance to cis-platinum	Zhu et al. (2022)
METTL3	LncRNA ARHGAP5-AS1	Cis-platinum	Enhance the stability of ARHGAP5-mRNA	Increased resistance to cis-platinum	Zhu et al. (2019b)
	ARHGAP5-mRNA				

### Esophageal Cancer

Esophageal cancer is a malignant tumour with a high degree of malignancy and mortality (Jain and Dhingra, 2017; Miller et al., 2020). m6A methylation is an important epigenetic modification involved in the physiological and pathological mechanisms of cancer. However, its role in esophageal cancer remains unclear. Current studies show that m6A modification plays a complex role in the occurrence, development and biological function of esophageal cancer, and it is a research hotspot in epigenetics. m6A modification also has therapeutic potential as an early diagnostic marker and therapeutic target in esophageal cancer. METTL3 has been identified as a decisive inducer of cancer progression, which is up-regulated in esophageal cancer and promotes epithelial-mesenchymal transformation, invasion and migration by regulating miR-20a-5p expression and inhibiting NFIC transcription (Liang et al., 2021). Furthermore, METTL3 was attributed to altered m6A levels in esophageal cancer, and its upregulation was significantly associated with cancer progression. Moreover, the deletion of METTL3 induces the G2/M arrest of esophageal cancer cells via the P21 signalling pathway (Zou et al., 2021). Therefore, METTL3 is a potential target molecule in esophageal cancer treatment. ALKBH5, an m6A regulatory factor, is down-regulated in esophageal cancer. The overexpression of ALKBH5 inhibits esophageal cancer cell proliferation and promotes ESCC cell apoptosis (Li et al., 2021a). Liu et al. revealed a METTL14-miR-99a-5p-TRIB2 positive feedback loop in esophageal cancer that enhances tumour stem cell characterisation and drug resistance of ESCC cells. METTL14 has been shown to play an antitumour role through its N6‐methyladenosine modification function. In esophageal cancer, METTL14 downregulation eliminated the inhibitory effect of Mir-99a-5p on TRIB2 expression by blocking Mir-99a-5p maturation, which subsequently increased the radiation-resistance of ESCC (Liu et al., 2021). Using METTL14’s effect on radiation therapy, Li et al. analysed 15 m6A regulatory factors and identified three new molecular subtypes associated with clinical features and esophageal cancer prognosis. By constructing a protein-protein interaction network for the three novel molecular subtypes and analysing their related genes, eight potential drugs (such as gefitinib, nalatinib, and imatinib) that closely interacted with these genes were identified. This study provides a valuable reference for identifying potential targets and drugs for esophageal cancer treatment (Li et al., 2022b). Currently, studies on m6A modification are limited to the interaction mechanism of esophageal cancer, and there is a lack of research on drug therapy. Therefore, further studies are needed to promote the application of m6A modification in clinical practice, such as the combination of m6A with chemotherapy and immunotherapy.

## Role of N6-Methyladenosine Modification in Tumour Cell Apoptosis, Autophagy and Ferroptosis

m6A is one of the richest modifications that determine the fate of RNA. Currently, m6A modification is closely related to tumorigenesis and plays an important role in the fate of tumour cells, including tumour proliferation and metastasis, tumour cell apoptosis, autophagy and iron death. The abnormal levels of m6A modification during the progression of apoptosis, autophagy, ferroptosis, necrosis and pyroptosis have been detected in gastrointestinal tumours (Zhi et al., 2022). Apoptosis, a type of cell death, is closely related to m6A modification (Zhi et al., 2022), wherein it regulates apoptosis by regulating apoptosis-related gene expression, silencing methylation or demethylase genes and reducing YTHDF2-mediated transcripts (Liu et al., 2022c). For example, METTL3 inhibits the apoptosis of non-small cell lung cancer (NSCLC) cells by promoting miR-1246 maturation and down-regulating PEG3 expression levels (Huang et al., 2021). In a recent study of lncRNAs containing m6A, LNC942 was observed to directly recruit METTL14, a core member of the m6A methyltransferase complex, and associated with increased levels of m6A methylation modification in breast cancer cells. Further, the LNC942-METTL14-CXCR4/CYP1B1 signal axis accelerated cell proliferation and colony formation, and reduced cell apoptosis rate (Sun et al., 2020).

Autophagy is a degradation process involving the lysosomal cytoplasmic content and autophagy-associated (ATG) proteins and transcription factors. It is also closely influenced by different stimulators and inhibitors. Autophagy can promote the resistance of tumour cells to chemotherapy and enable tumour cells to survive (Levy et al., 2017). Various studies have revealed the potential correlation between m6A modification and autophagy mechanism. Kong et al. found that METTL14 expression was higher in pancreatic cancer tissues than in non-tumour tissues, with METTL14 downregulation increasing the sensitivity of pancreatic cancer cells to cisplatin. Compared with the control group, the apoptosis and autophagy of tumour cells were significantly enhanced after METT14 gene knockout (Kong et al., 2020a). YTHDF1 expression is an independent prognostic factor for patients with HCC. Multiple HCC models confirmed that YTHDF1 cannot inhibit the autophagy, growth and metastasis of HCC (Li et al., 2021b). These findings highlight the interaction between autophagy and m6A regulators, but the relationship between m6A and autophagy remains unclear.

Ferroptosis is a novel pro-inflammatory programmed cell death pathway that plays a key role in the clearance of malignant cells. It is caused by the inhibition of the xCT/GSH/GPX4 axis and characterised by iron hyperplasia, lipid peroxidation and the compression of mitochondrial membrane density (Mou et al., 2019). Current studies on ferroptosis modified with m6A have focused on the interference of reader and writer factors on lipid peroxidation or antioxidant enzymes. However, few studies have detected the level of m6A erasers and their correlation with abnormal ferroptosis execution in cancer cells. In NSCLC, METTL3 is involved in cisplatin-mediated ferroptosis *via* m6A enrichment in FSP1 mRNA (Song et al., 2021). Thus, the relationship between m6A modification and iron death suggests that targeting m6A to induce ferrous iron death could be a promising therapeutic strategy.

## Conclusion and Perspectives

m6A RNA modification has attracted attention in epigenetic research and is involved in many biological processes and disease progressions. From the perspective of epigenetics, m6A modification provides novel insights into the pathogenesis of many diseases, especially tumours. However, further studies are required to understand the dynamic nature of m6A RNA modification in post-transcriptional regulation (Chen and Wong, 2020). m6A RNA modification plays an important role in promoting or inhibiting the growth, proliferation, migration, invasion, specific metastasis, drug resistance and prognosis of digestive tumours through three effector factors, writers, erasers and readers. With increasing studies on the network mechanism of m6A modification regulation, the related mechanism of m6A modification on tumour drug resistance will be clarified (Qiu et al., 2022).

Multiple studies showed that m6A-modified regulatory factors are resistant to SOR in HCC. In CRC, OX, cisplatin and other drug resistance are observed. Resistant to gemcitabine, 5-FU and cisplatin are also observed in pancreatic cancer. Additionally, m6A plays an important role in the regulation of OX and cisplatin resistance in gastric cancer. However, these studies are preliminary, requiring more systematic studies. Furthermore, translational studies are needed to further clarify the use of m6A alone or in combination with other therapies for the treatment of digestive tumours.

Immunotherapy is a new cancer treatment strategy that has been widely used to treat various solid tumours, including various digestive tract tumours and other solid tumours (Kong et al., 2021; Lee et al., 2021). In recent years, promising progress has been made in tumour immunotherapy with m6A modification, among which, ICIs harness the patient’s immune system, offering a novel method of cancer treatment. However, immunotherapy, such as ICIs, also presents drug resistance in some patients. For example, from the perspective of an m6A “writer”, Wang et al. found that the deletion of methyltransferases METTL3 and METTL14 inhibits m6A modification and enhances pMR-MSI-L response to PD-1 therapy in patients with CRC and melanoma, significantly delaying tumour growth and prolonging patient survival Wang et al. (2020b). However, large-scale basic research is needed to further clarify the specific mechanism of m6A modification in immunotherapy.

m6A plays various roles in different tumour types, suggesting the complexity and diversity of m6A modifications in drug resistance. Recent studies also showed that m6A-modified regulatory factors have potential as therapeutic targets and can enhance the sensitivity of tumour cells to anticancer drugs, providing a new research direction in solving the problem of anticancer drug resistance. This review also highlights the positive prospects of targeted m6A modifications.

## Data Availability

The original contributions presented in the study are included in the article/supplementary material, further inquiries can be directed to the corresponding author.
